# A generalized covariate-adjusted top-scoring pair algorithm with applications to diabetic kidney disease stage classification in the Chronic Renal Insufficiency Cohort (CRIC) Study

**DOI:** 10.1186/s12859-023-05171-w

**Published:** 2023-02-20

**Authors:** Brian Kwan, Tobias Fuhrer, Daniel Montemayor, Jeffery C. Fink, Jiang He, Chi-yuan Hsu, Karen Messer, Robert G. Nelson, Minya Pu, Ana C. Ricardo, Hernan Rincon-Choles, Vallabh O. Shah, Hongping Ye, Jing Zhang, Kumar Sharma, Loki Natarajan

**Affiliations:** 1 Division of Biostatistics and Bioinformatics, Herbert Wertheim School of Public Health, University of California, San Diego, La Jolla, CA USA; 2 Moores Cancer Center, University of California, San Diego, La Jolla, CA USA; 3 Institute of Molecular Systems Biology, ETH Zurich, Zurich, Switzerland; 4 Division of Nephrology, Department of Medicine, University of Texas Health San Antonio, San Antonio, TX USA; 5 Center for Renal Precision Medicine, University of Texas Health San Antonio, San Antonio, TX USA; 6 Department of Medicine, University of Maryland, Baltimore School of Medicine, Baltimore, MD USA; 7 Department of Epidemiology, Tulane University School of Public Health and Tropical Medicine and Tulane University Translational Science Institute,, New Orleans, LA USA; 8 Division of Nephrology, University of California, San Francisco School of Medicine, San Francisco, CA USA; 9 Chronic Kidney Disease Section, National Institute of Diabetes and Digestive and Kidney Diseases, Phoenix, AZ USA; 10 Department of Medicine, University of Illinois, Chicago, IL USA; 11 Department of Nephrology, Glickman Urological and Kidney Institute, Cleveland Clinic Foundation, Cleveland, OH USA; 12 University of New Mexico Health Sciences Center, Albuquerque, NM USA

**Keywords:** Biomarker, Classification, Feature selection, Kidney disease, Metabolomics, Order statistics, Ranking algorithm

## Abstract

**Background:**

The growing amount of high dimensional biomolecular data has spawned new statistical and computational models for risk prediction and disease classification. Yet, many of these methods do not yield biologically interpretable models, despite offering high classification accuracy. An exception, the top-scoring pair (TSP) algorithm derives parameter-free, biologically interpretable single pair decision rules that are accurate and robust in disease classification. However, standard TSP methods do not accommodate covariates that could heavily influence feature selection for the top-scoring pair. Herein, we propose a covariate-adjusted TSP method, which uses residuals from a regression of features on the covariates for identifying top scoring pairs. We conduct simulations and a data application to investigate our method, and compare it to existing classifiers, LASSO and random forests.

**Results:**

Our simulations found that features that were highly correlated with clinical variables had high likelihood of being selected as top scoring pairs in the standard TSP setting. However, through residualization, our covariate-adjusted TSP was able to identify new top scoring pairs, that were largely uncorrelated with clinical variables. In the data application, using patients with diabetes (n = 977) selected for metabolomic profiling in the Chronic Renal Insufficiency Cohort (CRIC) study, the standard TSP algorithm identified (valine-betaine, dimethyl-arg) as the top-scoring metabolite pair for classifying diabetic kidney disease (DKD) severity, whereas the covariate-adjusted TSP method identified the pair (pipazethate, octaethylene glycol) as top-scoring. Valine-betaine and dimethyl-arg had, respectively, ≥ 0.4 absolute correlation with urine albumin and serum creatinine, known prognosticators of DKD. Thus without covariate-adjustment the top-scoring pair largely reflected known markers of disease severity, whereas covariate-adjusted TSP uncovered features liberated from confounding, and identified independent prognostic markers of DKD severity. Furthermore, TSP-based methods achieved competitive classification accuracy in DKD to LASSO and random forests, while providing more parsimonious models.

**Conclusions:**

We extended TSP-based methods to account for covariates, via a simple, easy to implement residualizing process. Our covariate-adjusted TSP method identified metabolite features, uncorrelated from clinical covariates, that discriminate DKD severity stage based on the relative ordering between two features, and thus provide insights into future studies on the order reversals in early vs advanced disease states.

**Supplementary Information:**

The online version contains supplementary material available at 10.1186/s12859-023-05171-w.

## Background

The ever increasing amount of high-dimensional biomolecular data generated using high-throughput technologies has brought a critical need for decision rules that would strengthen our understanding of clinical diseases and health outcomes [1–4]. A prominent challenge is deriving decision rules that are not only accurate and robust across a diverse range of settings but also have ease of biological interpretability for a desired future clinic usage. Modern statistical and machine learning methods permeate the literature and frequently achieve high classification accuracy [5–8]. However, decision rules that make accurate assessments of patient disease outcomes may do so often at the expense of using nonlinear functions of hundreds or even thousands of features, which involves estimating a plethora of model parameters. This leads to the construction of highly complex decision boundaries for distinguishing between different classes of patients, which can be difficult to interpret and characterize in a biologically meaningful manner.

We focus on the parameter-free Top-Scoring Pair (TSP) algorithm [9], that has the advantage of providing simple and biologically interpretable decision rules. As a primer, the TSP algorithm identifies a single pair of features that best discriminates between two classes of interest among all possible feature-pairs—the top-scoring pair—along a predefined fixed decision boundary, a 45-degree line passing through the origin in the space defined by the two features. Measure of discrimination of a feature-pair is assessed via a score for which an observed ordering of the two features is more prominent in one class than in the other. After the pair of features with the maximal score is identified, classification entails assigning a test sample to the class for which the test sample’s ordering of the top-scoring pair is most common. As the TSP algorithm is concerned with the ordering of features, the method examines the ranks of the features within individual profiles prior to identifying the top-scoring pair. Since TSP bases selection in a ranks context, the algorithm is highly robust to data normalization procedures involving monotonic transformation of raw feature values.

The TSP algorithm has been noted for identifying gene-pair markers for diverse human disease classification, e.g., cancer, diabetes, and HIV, comparable to standard classification methods, while using much fewer genes [9, 10]. The K-TSP classifier, based on the top $k$ gene pairs and a majority voting procedure for classification, had competitive binary and multi-class prediction accuracy compared to TSP and standard methods [11]. In addition, integrating methods such as the support vector machine with the K-TSP algorithm can improve classification performance [12]. However, the existing TSP methods do not take into account possible covariates that influence the features, e.g., confounding clinical risk factors, in identifying the top-scoring pair and we aim to address this gap.

In this study, we extend the existing TSP and K-TSP methods [9, 13] by using the residuals from a regression of features on covariates of interest to select the top-scoring pairs, which differs from the typical practice of using the raw values of features. We demonstrate that our covariate-adjusted TSP method selects features largely liberated from the confounding effects of the covariates, and would thus select a top-scoring pair that discriminates outcome classes without influence from known covariates. We conduct a simulation study to illustrate the implications of using the features’ residuals as the input to the TSP algorithm. Furthermore, we demonstrate the application of our extended TSP and K-TSP methods to the novel setting of metabolomics and chronic kidney disease (CKD) in patients with diabetes mellitus enrolled in the Chronic Renal Insufficiency Cohort (CRIC) study. Recent reviews highlighted key metabolites that differentiated cases of diabetic kidney disease (DKD) from healthy controls [14–19] and thus we aim to identify top-scoring pairs of metabolite ions that best discriminate between severity stages of DKD using a diverse subsample of the CRIC study. Finally, we compare classification accuracies of TSP and K-TSP to popular statistical learning methods, i.e., LASSO (least absolute shrinkage and selection operator) and random forests [20].

## Methods

### TSP and K-TSP: brief review

We briefly review TSP and K-TSP methods. Let $X = \{ X_{1} , X_{2} , \ldots , X_{p}$} denote the $p$ features (e.g., metabolites) for an individual profile. The TSP algorithm [9] identifies the top-scoring feature pair ${\Theta }^{*}$ among the $p$ features with the maximum absolute difference in the probability of $X_{i} < X_{j}$ between two classes of individuals, $C = 1, 2$. Specifically, we calculate the discriminant score of all possible feature pairs $\left( {i, j} \right) \in {\Theta }$:

$\hat{s}_{ij} = \left| {P\left( {X_{i} < X_{j} {|}C = 1} \right) - P\left( {X_{i} < X_{j} {|}C = 2} \right)} \right|$ and define ${\Theta }^{*}$ $= \arg max_{{\left( {i, j} \right) \in {\Theta }}} \hat{s}_{ij}$. These conditional probabilities are estimated using maximum likelihood from the sample proportions of the observed ordering $X_{i} < X_{j}$ between both classes. Accordingly, it is sufficient to know the ranks of features within individual profiles to obtain the scores for all feature-pairs $\hat{s}_{ij} , i,j = 1,2, \ldots ,p, i \ne j$. A feature-pair ($i, j$) achieves perfect discrimination when $\hat{s}_{ij} = 1$ and no discrimination when $\hat{s}_{ij}$ = 0. If multiple pairs achieve the top score, ties were broken with a secondary rank-score to select a single top-scoring pair [21].

Classification with TSP amounts to observing the ordering of the two features of the top-scoring pair ($i, j$) for a test sample. If in the training data $P\left( {X_{i} < X_{j} {|}C = 1} \right) <$ $P\left( {X_{i} < X_{j} {|}C = 2} \right)$, then if we observe $X_{i} < X_{j}$ TSP classifies the test sample as class $C = 2$ or if in the test sample $X_{i} \ge X_{j}$ TSP classifies as class $C = 1$. Otherwise, if $P\left( {X_{i} < X_{j} {|}C = 1} \right) \ge P\left( {X_{i} < X_{j} {|}C = 2} \right)$ in the training data, then if we observe $X_{i} < X_{j}$ TSP classifies the test sample as class $C = 1$ or if instead observed as $X_{i} \ge X_{j}$ TSP classifies as class $C = 2$.

In practice, the results of TSP may be sensitive to perturbations in the training data and a more stable alternative, the K-TSP algorithm [11], may be useful. K-TSP defines the set of $k$ disjoint features pairs with the highest scores to be ${\Theta }_{{\text{k}}} = \{ \left( {i_{1} ,j_{1} } \right), \ldots , (i_{k} ,j_{k} )\}$. The set of $k$ disjoint top-scoring pairs is chosen ${\Theta }_{k}^{*} = \{ \left( {i_{1}^{*} ,j_{1}^{*} } \right), \ldots , (i_{k}^{*} ,j_{k}^{*} )\}$ to maximize the score $\hat{s}_{{i_{r} j_{r} }}$: ${\Theta }_{k}^{*} = \arg max_{{{\Theta }_{{\text{k}}} }} \mathop \sum \limits_{r = 1}^{k} \hat{s}_{{i_{r} j_{r} }}$ for each value $k$. From this, we now obtain the optimal value $k^{*}$ from the set of $k$ values that maximizes the following criterion $\hat{\tau }_{KTSP}$ in (1) motivated by the concept of analysis of variance [13]:

1 $$\begin{array}{*{20}c} {\hat{\tau }_{KTSP} \left( {{\Theta }_{k}^{*} } \right) = \frac{{\mathop \sum \nolimits_{r = 1}^{k} \hat{s}_{{i_{r} j_{r} }} }}{{\sqrt {\widehat{Var}\left( {\mathop \sum \nolimits_{r = 1}^{k} \left[ {I\left( {X_{{i_{r}^{*} }} < X_{{j_{r}^{*} }} } \right)} \right]|C = 1} \right) + \widehat{Var}\left( {\mathop \sum \nolimits_{r = 1}^{k} \left[ {I\left( {X_{{i_{r}^{*} }} < X_{{j_{r}^{*} }} } \right)} \right]|C = 2} \right)} }}} \\ \end{array}$$

Classification with K-TSP amounts to observing the ordering of the $k$ top-scoring pairs $\{ (i_{1}^{*} ,j_{1}^{*} ), \ldots , (i_{k}^{*} ,j_{k}^{*} )\}$ and taking a simple majority voting rule for a test sample. That is, the test sample will be assigned to the class receiving the most votes.

We implemented the TSP and K-TSP algorithms from the “switchbox” R package [22] for our statistical analysis. Our optimal value $k^{*}$ was selected from a range of $k$ values 2 to 10 for the K-TSP algorithm.

### Covariate-adjusted TSP method by residualizing the features

For most chronic diseases, there are clinical risk factors known to be associated with the outcome of interest. The aforementioned TSP methods do not take into account the effects of such variables, $Z = \{ Z_{1} , Z_{2} , \ldots , Z_{q}$}, in selecting top-scoring pairs. Therefore, these top-scoring pairs may be strongly confounded by $Z$ and we seek to suppress the effects of these confounders in the top-scoring pair selection. Our goal is to identify top-scoring pairs conditional on covariate values, $Z$:

2 $$\begin{array}{*{20}c} {\hat{s}_{ij} \left| {Z = } \right|P\left( {(X_{i} {|}Z{)} < (X_{j} {|}Z{)|}C = 1} \right) - P\left( {(X_{i} {|}Z{)} < (X_{j } {|}Z{)|}C = 2} \right)} \\ \end{array}$$

To operationalize this approach (2), we fit linear regression models with features $X$ as outcomes and $Z$ as covariates, and use the model residuals as opposed to feature values for selecting the top-scoring pairs. We refer to this data preprocessing step as residualizing which largely decorrelates features, $X$, from the individual covariates, $Z$. In particular, the fitted regression model for feature $X_{i} = \left\{ {X_{i1} , X_{i2} , \ldots , X_{iN} } \right\}$, such that $i = 1,2, \ldots ,p$, is defined as $\hat{X}_{ik} = \hat{\beta }_{0} + Z_{k1} \hat{\beta }_{i1} + Z_{k2} \hat{\beta }_{i2} + \ldots + Z_{kq} \hat{\beta }_{iq}$ for the $k$th individual, $k = 1,2, \ldots ,N$. We define the residual of the $i$th feature for the $k$th individual to be $e_{ik} = X_{ik} - \hat{X}_{ik}$ in which $e_{i} = \{ e_{i1} , e_{i2} , \ldots , e_{iN}$} is the set of residuals of the $i$th feature.

For the context of this paper, we distinguish between two types of features data. The features $X_{i}$ is the data as is and are designated as raw features and the features $e_{i}$ are the residuals of $X_{i}$ obtained from the residualizing process and are named as residualized features. Thus, two types of TSP-based methods were developed and compared in our simulations and application setting for feature selection and classification accuracy: (1) non-residualized, trained from the raw $X_{i}$, and (2) residualized, trained from the residualized $e_{i}$.

### Simulation setup

To evaluate the performance of the residualizing process on selection of the top-scoring pair, we conducted two simulation studies. For the first simulation study, we compared the effect of residualizing feature pairs that predict the outcome (e.g., disease status) through a covariate (e.g., clinical characteristic) versus features pairs that are independent of said covariate. For the second simulation study, we evaluated the impact of the correlation in these feature pairs on their selection as a top-scoring pair for both raw and residualized features.

In our first simulation study, we generated feature pairs with strong vs no correlation with covariate data, which itself is highly predictive of the outcome. Sample size was set to $N$ = 200 for a binary two class outcome ($Y$) with values 0 or 1. We considered the simple case of a single binary (0 or 1) covariate ($Z$) with a 50% population prevalence. We defined the high association between $Z$ and $Y$ as the probabilistic relationship $P\left( {Y = 1{|}Z} \right) = \left| {Z - 0.05} \right|$. Two different sets of bivariate data were generated to illustrate how residualizing impacts the scores of these feature pairs which, in turn, affects whether a feature pair is still likely to be selected as a top-scoring pair after residualizing.

The first set generated was bivariate normal $(X_{1} , X_{2} )$ conditional on $Z$:

$$\left( {\begin{array}{*{20}c} {X_{1} } \\ {X_{2} } \\ \end{array} } \right) |_{Z = 0} \sim N\left[ {\left( {\begin{array}{*{20}c} 0 \\ 5 \\ \end{array} } \right), \left( {\begin{array}{*{20}c} 2 & 0 \\ 0 & 2 \\ \end{array} } \right)} \right]; \left( {\begin{array}{*{20}c} {X_{1} } \\ {X_{2} } \\ \end{array} } \right) |_{Z = 1} \sim N\left[ {\left( {\begin{array}{*{20}c} 5 \\ 0 \\ \end{array} } \right), \left( {\begin{array}{*{20}c} 2 & 0 \\ 0 & 2 \\ \end{array} } \right)} \right]$$

The second set was also bivariate normal $(X_{3} , X_{4} )$ but conditional on $Y$ and independent of $Z$:

$$\left( {\begin{array}{*{20}c} {X_{3} } \\ {X_{4} } \\ \end{array} } \right) |_{Y = 0} \sim N\left[ {\left( {\begin{array}{*{20}c} 0 \\ {2.5} \\ \end{array} } \right), \left( {\begin{array}{*{20}c} 3 & 0 \\ 0 & 3 \\ \end{array} } \right)} \right]; \left( {\begin{array}{*{20}c} {X_{3} } \\ {X_{4} } \\ \end{array} } \right) |_{Y = 1} \sim N\left[ {\left( {\begin{array}{*{20}c} {2.5} \\ 0 \\ \end{array} } \right), \left( {\begin{array}{*{20}c} 3 & 0 \\ 0 & 3 \\ \end{array} } \right)} \right]$$

With features $(X_{1} , X_{2} , X_{3} , X_{4} )$ and covariate data $Z$, we evaluated the posterior probabilities of $Y$ as $P\left( {Y{|}X_{1} , X_{2} , X_{3} , X_{4} , Z} \right)$ using Bayes’ theorem, noting that $(X_{1} , X_{2} )$ is conditionally independent of $Y$:

$$P\left( {Y{|}X_{1} , X_{2} , X_{3} , X_{4} , Z} \right) = P\left( {Y{|}X_{3} ,X_{4} , Z} \right) = \frac{{P\left( {X_{3} ,X_{4} {|}Y, Z} \right)*P(Y|Z)}}{{P(X_{3} ,X_{4} |Z)}}$$

These posterior probabilities constituted Bernoulli probabilities, and were used to generate the binary outcome $Y$. Finally, we calculated the TSP scores for the raw and residualized (on $Z$) variants of $(X_{1} , X_{2} )$ and $(X_{3} , X_{4} )$.

In our second simulation study, we generated the feature pairs data similar to the first study except we consider a range of correlation values of $(X_{1} , X_{2} )$ and $(X_{3} , X_{4} )$. In particular, we consider a grid of correlation values from -1 to 1 in increments of 0.01. For each correlation value $\rho$, we generate our feature pairs as

$$\begin{aligned} & \left( {\begin{array}{*{20}c} {X_{1} } \\ {X_{2} } \\ \end{array} } \right) |_{Z = 0} \sim N\left[ {\left( {\begin{array}{*{20}c} 0 \\ 5 \\ \end{array} } \right), \left( {\begin{array}{*{20}c} 2 & {2\rho } \\ {2\rho } & 2 \\ \end{array} } \right)} \right]; \quad \left( {\begin{array}{*{20}c} {X_{1} } \\ {X_{2} } \\ \end{array} } \right) |_{Z = 1} \sim N\left[ {\left( {\begin{array}{*{20}c} 5 \\ 0 \\ \end{array} } \right), \left( {\begin{array}{*{20}c} 2 & {2\rho } \\ {2\rho } & 2 \\ \end{array} } \right)} \right] \\ & \left( {\begin{array}{*{20}c} {X_{3} } \\ {X_{4} } \\ \end{array} } \right) |_{Y = 0} \sim N\left[ {\left( {\begin{array}{*{20}c} 0 \\ {2.5} \\ \end{array} } \right), \left( {\begin{array}{*{20}c} 3 & {3\rho } \\ {3\rho } & 3 \\ \end{array} } \right)} \right]; \quad \left( {\begin{array}{*{20}c} {X_{3} } \\ {X_{4} } \\ \end{array} } \right) |_{Y = 1} \sim N\left[ {\left( {\begin{array}{*{20}c} {2.5} \\ 0 \\ \end{array} } \right), \left( {\begin{array}{*{20}c} 3 & {3\rho } \\ {3\rho } & 3 \\ \end{array} } \right)} \right] \\ \end{aligned}$$

After assigning the binary outcomes via posterior probabilities of $Y$, we calculated the TSP scores for the raw and residualized (on $Z$) variants of $(X_{1} , X_{2} )$ and $(X_{3} , X_{4} )$ at the correlation value $\rho$.

### CRIC study sample

We illustrate a real data application of our extended TSP and K-TSP methods to a subsample of the CRIC study. The parent CRIC study [23–25] included a racially and ethnically diverse group of adults aged 21–74 years with estimated glomerular filtration rate (eGFR) between 20 and 70 ml/min/1.73 m^2^ at baseline, and a broad spectrum of kidney disease severity.

Our study sample consisted of 977 CRIC participants with diabetes selected for untargeted metabolome profiling [26]. These participants had complete data on the baseline characteristics age, race, sex, smoked > 100 cigarettes in lifetime, body mass index (BMI), hemoglobin A1c (HbA1c), mean arterial pressure, urine albumin, serum creatinine, and angiotensin-converting enzyme (ACE) inhibitor or angiotensin-receptor blockers (ARB) use, which are the selected covariates for our residualizing process. Baseline characteristics of our CRIC study sample are displayed in Table 1.

**Table 1:** Baseline characteristics of our CRIC study sample (N = 977) selected for untargeted metabolome profiling

Age (years)	59.94 ± 9.43
*Race*	
White	436 (45)
Black	410 (42)
Other	131 (13)
*Sex*	
Male	551 (56)
Female	426 (44)
*Smoked* > *100 cigarettes*	
Yes	558 (57)
No	419 (43)
BMI (kg/m^2^)	34.18 ± 7.94
HbAlc (%)	7.57 ± 1.55
Mean arterial pressure (mmHg)	89.85 ± 13.26
Urine albumin (g/24 h)	0.92 ± 1.81
Serum creatinine (mg/dL)	1.92 ± 0.6
*ACE Inhibitor or ARB use*	
Yes	790 (81)
No	187 (19)
eGFR (ml/min/1.73^2^)	40.61 ± 11.17

Values are expressed as mean ± SD or N (%)

CRIC, Chronic Renal Insufficiency Cohort; BMI, body mass index; HbA1c, hemoglobin A1c; ACE, angiotensin-converting enzyme; ARB, angiotensin-receptor blocker; eGFR, estimated glomerular filtration rate

Participants were sampled across CKD stages G2 (eGFR 60–70), G3a (eGFR 45–60), G3b (eGFR 30–45) and G4 (eGFR 20–30). The outcome for our analysis is a binary indicator of early-stage DKD (stage G2-3b, N = 777) versus advanced-stage DKD (stage G4, N = 200). Participants with early-stage DKD had mean (SD) eGFR 44.7 (8.5) ml/min/1.73 m^2^, while those with advanced-stage DKD had mean (SD) eGFR 24.8 (3.6) ml/min/1.73 m^2^.

### Metabolomics

Untargeted metabolome profiling in urine was performed for our 977 CRIC samples. Assay procedures have been described previously [26, 27], but we briefly recapitulate key points here for completeness. Aliquots of urine samples stored at -80 °C and limited to less than 3 free thaw cycles were used. Relative metabolite ion abundance was quantified with a MPS3xt autosampler (Gerstel) coupled to an Agilent 6550 Q-TOF mass spectrometer (Agilent Technologies) by non-targeted flow injection analysis [27]. Profile mass spectra were recorded in 4Ghz acquisition mode from 50 to 1000 m/z in negative ionization mode. Raw mass spectrometry data was normalized based on creatinine ion abundances. Final annotation of ions was based on accurate mass comparison using 1 mDa mass tolerance against Human Metabolome Database HMDBv4.0 assuming single deprotonation. We consider a final set of 698 annotated metabolite ions for our analysis. A single ion could annotate multiple metabolites resulting in ambiguities in the assignments. Therefore, we shall refer to our features as metabolite ions for our study.

As described in “Covariate-adjusted TSP method by residualizing the features" section, we consider raw metabolite ions comprising the creatinine-normalized abundances as is without residualizing, and the residualized metabolite ions comprising the residuals of regressing each creatinine-normalized ion on the aforementioned covariates.

### Comparison to other methods: LASSO and random forests

To evaluate the relative prediction performance of the TSP and K-TSP algorithms for DKD severity, we compared these methods to LASSO and random forests. The LASSO model was tuned to the regularization parameter that minimizes mean tenfold cross-validated misclassification error for feature selection among the 698 metabolite ions [28]. The random forests model was fitted using Breiman’s algorithm, growing 500 trees and randomly sampling the square root of the total number of available variables ($\sqrt {698} \approx 26$) as candidates at each split [29]. We implemented the LASSO and random forest methods using the R packages glmnet and randomForest, respectively.

Several classification accuracy measures were used for comparing TSP, K-TSP, LASSO, and random forests: (1) overall accuracy, i.e., overall proportion correctly classified, (2) sensitivity, i.e., proportion correctly classified among those with advanced-stage DKD, (3) specificity, i.e., proportion correctly classified among those with early-stage DKD, (4) balanced accuracy, i.e., average of sensitivity and specificity, (5) positive predictive value (PPV), i.e. proportion that truly have advanced-stage DKD among those classified with advanced-stage DKD, and (6) negative predictive value (NPV), i.e. proportion that truly have early-stage DKD among those classified with early-stage DKD. To gauge the variability in the measures, we conducted one-hundred iterations of fivefold cross-validation for each of these accuracy measures. One fold is held out as a test set and our models are trained on the remaining four folds with our accuracy measures calculated on the test set. The four folds in the training data will also each serve as a test set, which would result in accuracy measures from all five folds. The averages of these accuracy measures across all five folds are our fivefold cross validated estimates. Since the partition of the five folds varies for each iteration, TSP, K-TSP, LASSO, and random forests may select different metabolite ion predictors at each iteration.

## Results

### Simulation results

**Fig. 1:**
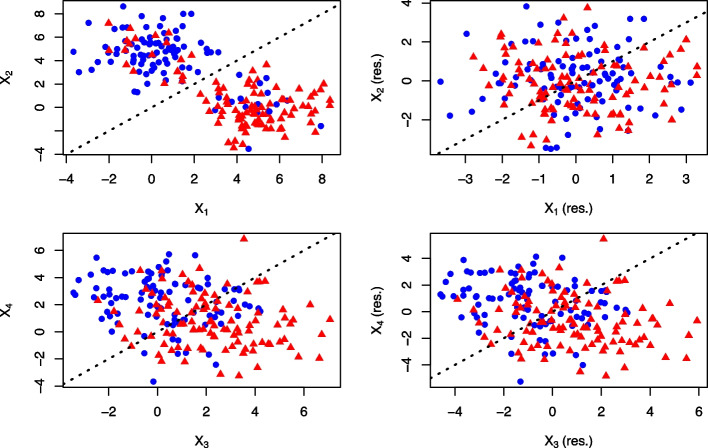
Comparison of residualizing feature pairs from the first simulation study. Left column: Scatter plots of generated feature pairs from our first simulation study (N = 200) conditional on our single “clinical” covariate, $(X_{1} ,{ }X_{2} )$, and independent of our single “clinical” covariate, $(X_{3} ,{ }X_{4} )$. Right column: Scatter plots of the residualized feature pairs. The two evenly split classes are represented as red and blue and TSP’s decision boundary is overlayed on the plots

The results of our first simulation study are plotted in Fig. 1 with the rows corresponding to $(X_{1} , X_{2} )$ and $(X_{3} , X_{4} )$ and columns to their raw and residualized features. In total, 96 samples had class $Y = 0$ (48%) and 104 samples had class $Y = 1$ (52%). Both classes of $Y$ from the raw $(X_{1} , X_{2} )$ data were mostly well separated by the TSP’s fixed decision boundary and this raw pair had a score of 0.64. However, the residualized $(X_{1} , X_{2} )$ exhibited inadequate discrimination of $Y$ evident in the much lower score of 0.07. We can attribute this to the raw $(X_{1} , X_{2} )$ generated conditional on $Z$ which is strongly associated with $Y$. Residualizing the raw $(X_{1} , X_{2} )$ decorrelates the pair from $Z$, which substantially decreases the capability of $(X_{1} , X_{2} )$ to discriminate between the two classes of $Y$ along TSP’s decision boundary. In practice, if the raw values of $(X_{1} , X_{2} )$ are identified as the top-scoring pair, and we know this pair to be highly dependent on $Z$, then residualizing $(X_{1} , X_{2} )$ with $Z$ would most likely drop its candidacy as a top-scoring pair and opens the door for another feature pair to be selected for best discriminating between the classes of $Y$.

In contrast, residualizing the raw $(X_{3} , X_{4} )$ with $Z$ did not drastically affect the feature pair’s capability to distinguish between both classes of $Y$ along the decision boundary based on the small drop in score from 0.38 to 0.33. We can attribute this to the raw $(X_{3} , X_{4} )$ generated conditional on $Y$ and independent on $Z$, which would likely retain $(X_{3} , X_{4} )$ as the top-scoring pair even after residualizing with $Z$. In summary, residualizing captures top-scoring pairs liberated from the extraneous influence of covariates, which helps to identify potentially novel markers of outcome.

**Fig. 2:**
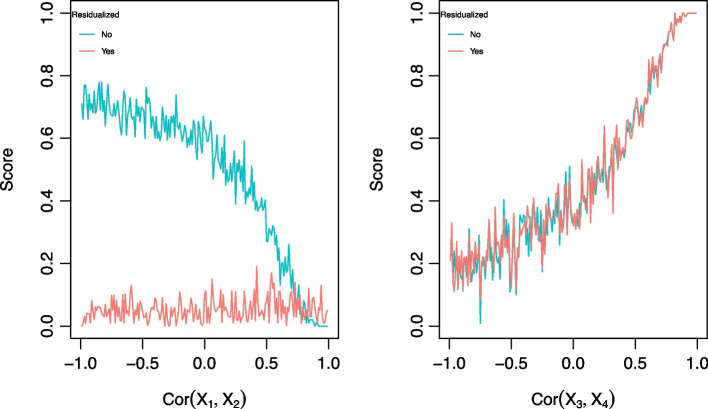
TSP score varying by correlation in feature pairs from the second simulation study. Left: Varying the correlation of $(X_{1} , X_{2} )$, i.e., pair conditional on our single “clinical” covariate. Right: Varying the correlation of $(X_{3} , X_{4} )$, i.e., pair independent of our single “clinical” covariate

The results of our second simulation study are plotted in Fig. 2. For the raw $(X_{1} , X_{2} )$, increasing correlation values corresponds to a score decline. Meanwhile, the residualized $(X_{1} , X_{2} )$ has low scores across the range of correlation values, not exceeding 0.2. Regardless of how correlated $X_{1}$ is to $X_{2}$, residualizing $(X_{1} , X_{2} )$ with $Z$ would most likely eliminate it as a top-scoring pair. Interestingly, the raw and residualized $(X_{3} , X_{4} )$ had similar scores with increasing values as correlation between the features increased. Residualizing the raw $(X_{3} , X_{4} )$ with $Z$ did not have a major effect on the feature pair’s capability to distinguish between the classes of $Y$. Thus even for (highly) correlated feature pairs, the residualizing process can identify novel markers of outcome liberated from the influence of covariates.

### TSP and K-TSP results on CRIC Study with and without residualizing

We apply the TSP and K-TSP algorithms to our CRIC study sample to identify metabolite ion pairs that best discriminate between DKD stage severity (early-stage vs advanced-stage), with and without residualizing the metabolite ions. Since the use of TSP is relatively novel in metabolomics studies, we provide a further extensive evaluation of the TSP algorithm based on discriminating urine samples of patients with type 2 diabetes mellitus from those of healthy controls on an independent data set in the Additional file 1. Here, we focus on the results from our CRIC study sample as the CRIC study contains one of the largest cohorts of individuals with diabetes in the US, with comprehensive data on metabolite and clinical profiles.

**Fig. 3:**
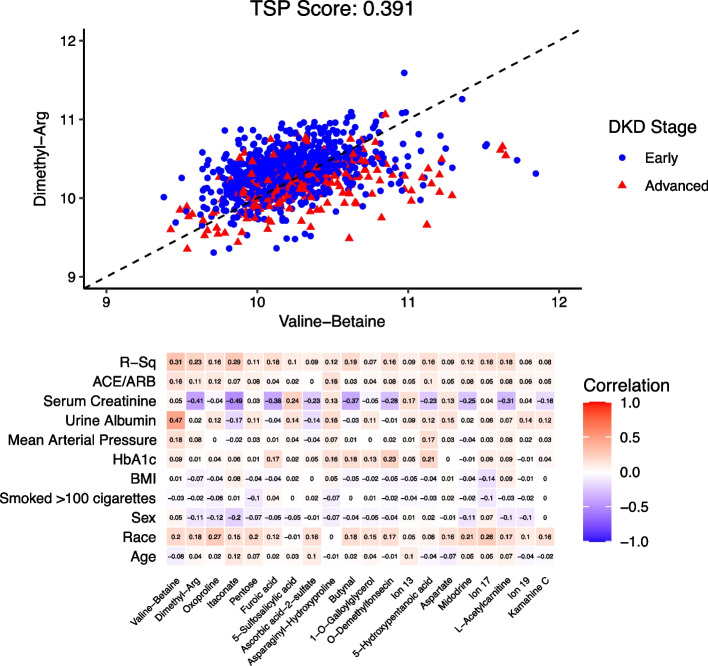
Top-scoring pairs for discriminating DKD stage from among the raw metabolite ions. **a** Scatter plot for the top pair of raw metabolite ions selected by the TSP algorithm along with TSP’s decision boundary. The axes are metabolite ion abundances that were creatinine normalized and natural log transformed. Patients had either early-stage DKD (stage G2-3b, N = 777) or advanced-stage DKD (stage G4, N = 200). **b** Heatmap correlation matrix of clinical variables vs raw metabolite ions selected by the K-TSP algorithm. Single ion can annotate to multiple metabolites, which resulted in ambiguity in assignments. Metabolite ion (full annotated name): Ion 13: 3,6-Dihydro-4-(4-methyl-3-pentenyl)-1,2-dithiin. Ion 17: 13,14,15-trihydroxy-9-oxo-8,17-dioxatetracyclo[8.7.0.02,7.011,16]heptadeca-1(10),2(7),3,5,11,13,15-heptaen-5-yl acetate. Ion 19: 2-Phenylethyl beta-d-glucopyranoside

From among the raw metabolite ions, the TSP algorithm identified the metabolite ion pair (annotated as valine-betaine, dimethyl-arg) to be the top-scoring pair (score: 0.391) in Fig. 3a. As mentioned earlier, a single ion could be annotated as multiple metabolites; the selected top-scoring pair contained an ion that could be annotated as valine or betaine, henceforth referred to as valine-betaine. Here, the TSP’s decision rule is that if a test patient’s observed raw metabolite ion ordering is valine-betaine < dimethyl-arg then the patient will be classified as having early-stage DKD and the reversed ordering for advanced-stage DKD. Applying the K-TSP algorithm gave us a total of 10 metabolite ion pairs, including (valine-betaine, dimethyl-arg), with score range 0.259–0.391. These 20 metabolite ions are listed in the correlation heatmap with the clinical variables used for the residualizing process in Fig. 3b. The metabolite ions valine-betaine and dimethyl-arg have the largest variation explained by the clinical variables with $R^{2}$ values 0.31 and 0.23, respectively. Notably, valine-betaine has a relatively high correlation with urine albumin (0.47) and dimethyl-arg a negative correlation for serum creatinine (-0.41), which likely indicates that in this application, TSP selected metabolites ions with a moderate-high correlation with known clinical markers of kidney disease.

**Fig. 4:**
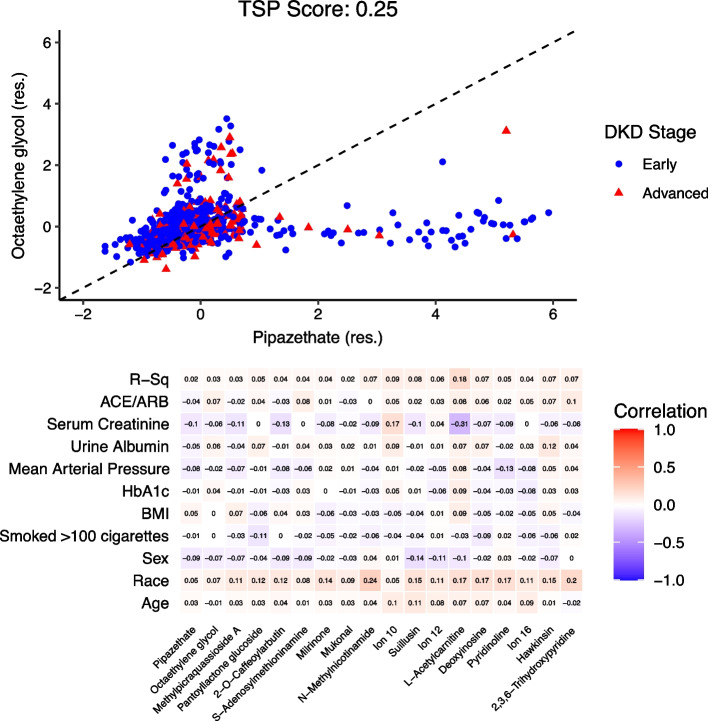
Top-scoring pairs for discriminating DKD stage from among the residualized metabolite ions. **a** Scatter plot for the top pair of residualized metabolite ions selected by the TSP algorithm along with TSP’s decision boundary. The axes are residuals of metabolite ion abundances that were creatinine normalized and natural log transformed. Patients had either early-stage DKD (stage G2-3b, N = 777) or advanced-stage DKD (stage G4, N = 200). **b** Heatmap correlation matrix of clinical variables vs the raw values of residualized metabolite ions selected by the K-TSP algorithm. Single ion can annotate to multiple metabolites, which resulted in ambiguity in assignments. Metabolite ion (full annotated name): Ion 10: 3,6-Dihydro-4-(4-methyl-3-pentenyl)-1,2-dithiin. Ion 12: [4-(5-hydroxy-7-methoxy-8-methyl-4-oxo-4H-chromen-3-yl)-2-methoxyphenyl]oxidanesulfonic acid. Ion 16: alpha-L-Rhamnopyranosyl-(1- > 3)-alpha-d-galactopyranosyl-(1–> 3)-L-fucose

After residualizing ions, the TSP algorithm instead identified the metabolite ion pair (pipazethate, octaethylene glycol) to be the top-scoring pair (score: 0.25). This TSP’s decision rule is that if a test patient’s observed residualized metabolite ion ordering is pipazethate < octaethylene glycol then the patient will be classified as having early-stage DKD and the reversed ordering for advanced-stage DKD in Fig. 4a. Applying the K-TSP algorithm gave us a total of 9 metabolite ion pairs, including (pipazethate, octaethylene glycol), with score range 0.158–0.25. These 18 metabolite ions are listed in the correlation heatmap with the clinical variables used for the residualizing process in Fig. 4b. The $R^{2}$ values for these 18 metabolite ions are much smaller than those of the 20 metabolite ions selected under the raw metabolomics setting. Here, pipazethate and octaethylene glycol do not have relatively high correlation values with the clinical variables indicating that these metabolite ions are not serving as proxies for clinical markers of kidney disease.

### Comparing TSP and K-TSP to LASSO and random forests

The application of LASSO to our full CRIC study sample identified 127 raw metabolite ions that best discriminate DKD severity stages, but zero residualized metabolite ions. To compare LASSO’s top metabolite ions with the 20 selected by K-TSP, we list the top 20 selected raw and residualized metabolite ion predictors by effect size for LASSO. Similarly, we selected the top 20 for random forests by mean decrease in Gini index from splitting on the metabolite ion, averaged over 500 trees, in the Supplementary Material. Three of LASSO’s top 20 raw metabolite ions were also selected under the raw setting for K-TSP. Meanwhile, random forests selected both metabolite ions of the raw TSP (valine-betaine, dimethyl-arg) in the top 20, with valine-betaine for the raw and residualized settings and dimethyl-arg only for the raw setting. In addition, random forests had one of the two metabolite ions in the residualized TSP, pipazethate, in the top 20 for the residualized setting.

**Fig. 5:**
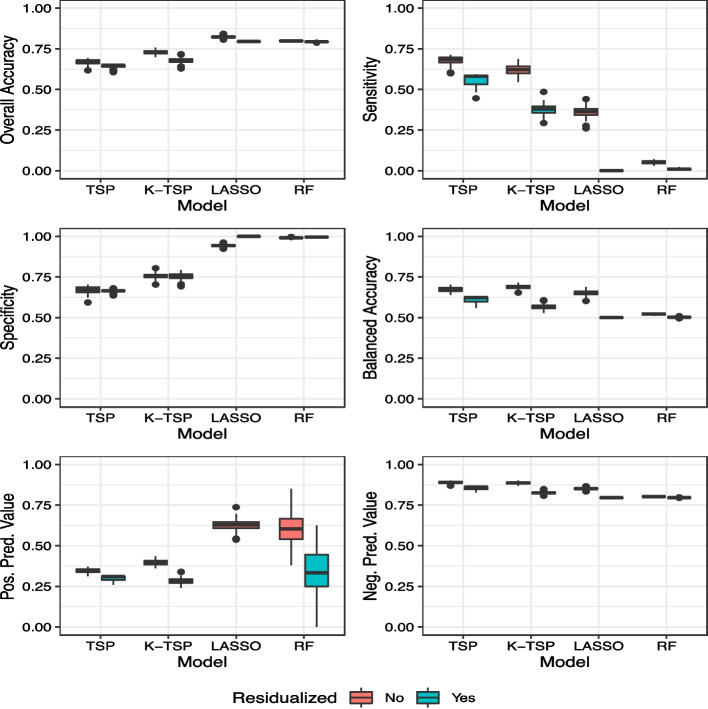
Model prediction results of TSP-based methods vs LASSO vs random forests. Box plots of model prediction performance for DKD stage: 100 iterations of fivefold cross-validated **a** overall accuracy, **b** sensitivity, **c** specificity, **d** balanced accuracy, **e** positive predictive value, and **f** negative predictive value. Cutoff value for prediction in LASSO and random forests is 0.5. Boxplot for LASSO using residualized metabolite ions not displayed in (**e**) because method did not predict advanced-stage DKD even once for any patient in all iterations. Model type: (K-)TSP: (K) Top-Scoring Pair(s). LASSO: Least Absolute Shrinkage and Selection Operator. RF: Random Forests

Results for overall accuracy, sensitivity, specificity, and balanced accuracy of early-stage DKD vs advanced-stage DKD for our statistical methods with type of metabolite ion predictors are displayed in Fig. 5. TSP and K-TSP had lower median cross-validated overall accuracy (0.649–0.728) compared to that of LASSO and random forests (0.793–0.823) with either raw or residualized metabolite ions. However, both LASSO and random forests displayed extremely poor sensitivity and high specificity, which indicates that the overall accuracy for these two methods is driven by classifying an overwhelmingly large number of patients as having early-stage DKD, regardless of their observed DKD stage. In contrast, TSP and K-TSP achieved a more balanced tradeoff of sensitivity and specificity and had values closer to their overall accuracy. Notably, we have imbalanced classes with 79.5% of our patients with early-stage DKD and we examine balanced accuracy for our methods which is preferred over overall accuracy for class imbalance data. Here, TSP and K-TSP did have higher median cross-validated balanced accuracy (0.566–0.689) compared to that of LASSO and random forests (0.5–0.652) for the raw or residualized metabolite ion cases. For positive predictive value, TSP and K-TSP did not perform better than LASSO or random forests when using raw metabolite ions; however, random forests displayed relatively high variability in its cross-validated values, and when using residualized metabolite ions, LASSO did not predict advanced-stage DKD even once for any patient in all 100 iterations of fivefold cross-validation (hence the absence of its boxplot in Fig. 5e). Finally, for negative predictive value, all methods performed reasonably well with TSP and K-TSP taking the lead. Therefore, TSP and K-TSP displayed comparatively good classification for patients with early-stage DKD from their specificity and NPV performances. Residualized metabolite ions are a valid option as predictors for our statistical methods in classification for patients with early-stage DKD. In particular, specificity and NPV did not show a notable decrease in performance going from using raw metabolite ions to their residualized variants while taking into account that the residualized metabolite ions are features much liberated from the effects of confounding clinical variables known to be associated with DKD severity.

Given the imbalanced classes with 20.5% of our sample having advanced-stage DKD, we also investigated classification results when lowering our previous optimal Bayes threshold of 0.5. For LASSO and random forests we instead use the sample prevalence (0.205) as a threshold for prediction (presented as a figure in the Supplementary Material). The lower cutoff yielded higher sensitivity for LASSO and random forests, which was previously a major concern in Fig. 5b. However, there was a decrease in specificity and PPV. As such, using a cutoff value to improve sensitivity is a trade-off and does not necessarily translate to better performance in other accuracy measures of interest. Importantly, the TSP methods are agnostic to the threshold used as it based on feature ordering and a fixed decision boundary, highlighting another advantage of using TSP methods.

## Discussion

The large influx of high-dimensional biomolecular data brought about by cutting-edge high-throughput technologies unleashes extraordinary potential for improving our understanding of the link between biological, in particular metabolic, profiles and clinical diseases. There is a critical need for accurate and robust decision rules based on these biological data that are easily interpretable for translation to future clinic use. We focused on the TSP (and K-TSP) algorithm that identifies a single pair (and set of pairs) of features that best discriminates between two classes of interest among all possible feature pairs. TSP identifies the feature pair for which an observed ordering of the two features is more common in one class than in the other, which allows the top-scoring pair to be interpreted as a key metabolic feature discriminating one class to another based on feature ordering. Hence, the TSP approach by construction aims to offer insights into underlying mechanisms of disease, a salient advantage over other statistical and machine learning methods.

A major potential of `omics studies is the possibility to discover “new” insights into disease mechanisms and discrimination; hence, interest usually lies in identifying markers that are associated with disease status after adjustment for known clinical factors. Previous studies utilizing TSP methods have not accounted for possible covariates in the selection of the top-scoring pairs. We provide an extension of the TSP algorithm for removing much of the extraneous effects that covariates could have on the features, so as to capture a top-scoring pair largely independent of covariates. We implement a residualizing process, and demonstrate via simulation and application that using the residuals from a regression of features on covariates known to be highly associated with the outcome, and then applying the TSP algorithm to these residuals, could identify potentially novel pairs compared to simply using the raw (unresidualized) features. In fact in our data application, the top-scoring pairs using the raw features were valine (or betaine) and dimethyl-arginine, known amino acids linked to albuminuria [18, 30], a potent risk factor for CKD. As a result, this pair could simply reflect a known underlying CKD marker, as seen in Fig. 3b. Conversely, the top-scoring pair from the residualized analysis were pipezethate [31], a non-narcotic antitussive agent, and octaethylene glycol [32–35], a member of the class of polyethylene glycols, found in osmotic laxatives. Thus these residualized metabolite ions, are potentially new markers, and could offer insights into drug metabolism and CKD. We note that these markers do not imply a causal link with disease outcome, but rather indicate reversals in marker ordering for disease stage states. Notably, the idea of using residuals to adjust for covariates has been considered in classical discriminant analysis [36–39], and more recently for decision trees [40]. Since this adjustment can be seen as a data preprocessing step; it can be applicable prior to any machine learning training step. However, to our knowledge, our use of residuals for the TSP algorithm is novel.

TSP and K-TSP are classification methods, hence we evaluated and compared their classification accuracy of DKD stage using metabolite ion predictors to more conventional statistical learning methods, i.e., LASSO and random forests. Based on the balanced accuracy metric, TSP and K-TSP outperform LASSO and random forests. Both TSP and K-TSP performed moderately and well in specificity and negative predictive value, respectively, suggesting that these methods can accurately identify a healthier or less severe disease group. Furthermore, using residualized metabolite ions yielded similar specificity and negative predictive value results for TSP and K-TSP.

We acknowledge limitations and future directions of our work. We evaluated a binary class outcome since the TSP and K-TSP algorithms were developed as binary classification methods. However, methods exist for multi-class classification [41, 42], which could be easily extended to our setting. In particular, for our DKD setting, multi-class would allow us to use residualized features to discriminate between different levels of kidney (dys)function among patients with diabetes; given the relatively small cell sizes we leave this to future work using a larger cohort. In addition, our residualizing process involves the use of linear regression to obtain the residuals of the features and more complex statistical models could be fitted to obtain the residuals, especially if there is notable evidence of heteroscedasticity. However, the linear regression models have simple implementation and have the residuals orthogonal to the covariates, which is beneficial for capturing cleaner features.

## Conclusion

In summary, in this work we extended the TSP-algorithms to account for clinical covariates, via a simple, easy to implement residualizing process. The TSP and K-TSP algorithms have the advantage of deriving parameter-free decision rules that best discriminate the class outcome of interest by examining just the ordering of feature pairs. Thus, they yield parsimonious classifiers that are biologically interpretable in the `omics setting. We demonstrated the utility of our residualizing approach for TSP via simulation and real application to the novel metabolite-DKD context. The residualized metabolite ion top-scoring pairs, being largely uncorrelated to clinical covariates, represent potentially independent markers for best discriminating disease stage. These metabolite ions could serve to motivate hypotheses for future studies, for instance, laboratory studies could further examine the selected pairs to confirm or refute the order reversals in the disease vs non-disease states.

## Supplementary Information

**Additional file 1**. Supplementary material.

## Data Availability

The datasets generated and/or analyzed during the current study are available upon request in the Chronic Renal Insufficiency Cohort Study Repository, https://repository.niddk.nih.gov/studies/cric/.
