# Supplementary material for: A generalized covariate-adjusted top-scoring pair algorithm with applications to diabetic kidney disease stage classification in the Chronic Renal Insufficiency Cohort (CRIC) Study
Source: BMC Bioinformatics. 2023 Feb 20;24:57. doi: 10.1186/s12859-023-05171-w (PMC9942303; doi:10.1186/s12859-023-05171-w)
Supplement: Supplementary file 1 — Additional file 1. Supplementary material. [file 12859_2023_5171_MOESM1_ESM.docx]

**Supplementary Materials for “A generalized covariate-adjusted top-scoring pair algorithm with applications to diabetic kidney disease stage classification in the Chronic Renal Insufficiency Cohort (CRIC) Study”**

**by Brian Kwan, Tobias Fuhrer, Daniel Montemayor, Jeffery C. Fink,**

**Jiang He, Chi-yuan Hsu, Karen Messer, Robert G. Nelson, Minya Pu, Ana C. Ricardo, Hernan Rincon-Choles, Vallabh O. Shah, Hongping Ye,**

**Jing Zhang, Kumar Sharma, and Loki Natarajan**

**Contents**

**A Further extensive evaluation of the TSP algorithm on a metabolomics study**

**B Top 20 metabolite ions predictors from the LASSO and random forests**

**C Box plots of model prediction performance for DKD stage using the sample prevalence of patients with advanced stage DKD as the cutoff value**

**A Further extensive evaluation of the TSP algorithm on a metabolomics study**

As mentioned in Section 3.2, we provide a further extensive evaluation of the TSP algorithm based on discriminating urine samples of patients with type 2 diabetes mellitus from those of healthy controls on an independent data set in the Supplementary Materials. Notably, the data is from a published paper (Salek RM, Maguire ML, Bentley E et al. A metabolomic comparison of urinary changes in type 2 diabetes in mouse, rat, and human. Physiol Genomics. 2007; 29(2):99-108. <https://doi.org/10.1152/physiolgenomics.00194.2006>) and is freely available at the web-accessible database MetaboLights. We illustrate our TSP application on this metabolomics study of 84 urine samples from healthy volunteers and 48 samples from patients with type 2 diabetes mellitus. Extensive details on the study are provided in the paper.

From among the raw metabolites, the TSP algorithm identified the metabolite pair (annotated as ornithine, isoleucine) to be the top-scoring pair (score: 0.58) shown in the immediate figure below.

Supplementary Figure 1: Scatterplot for the top-scoring pair for discriminating urine samples between healthy controls and patients with type 2 diabetes mellitus (disease) from among the raw metabolites along with TSP’s decision boundary. The axes are metabolite concentrations.

Here, the TSP’s decision rule is that if a sample’s observed raw metabolite ordering is ornithine < isoleucine then the sample will be classified as a healthy control and the reversed ordering for type 2 diabetes mellitus.

In addition, we applied our covariate-adjusted TSP method to residualize the metabolite features using sex as a covariate. After residualizing metabolites, the TSP algorithm instead identified the metabolite pair (2-oxoisovalerate, isoleucine) to be the top-scoring pair (score: 0.67) shown in the immediate figure below.

Supplementary Figure 2: Scatterplot for the top-scoring pair for discriminating urine samples between healthy controls and patients with type 2 diabetes mellitus (disease) from among the residualized metabolites along with TSP’s decision boundary. The axes are residuals of metabolite concentrations.

This TSP’s decision rule is that if a test patient’s observed residualized metabolite ordering is 2-oxoisovalerate < isoleucine then the patient will be classified as a healthy control and the reversed ordering for type 2 diabetes mellitus.

Based on this TSP application, we observe that covariate adjustment for TSP via residualizing process captures not only a different top-scoring pair compared to the raw metabolite setting, but also a pair with a higher score (0.67 > 0.58) which indicates that adjusting for sex identifies a novel sex-independent pair that improves on discriminating the healthy controls from type 2 diabetes mellitus. Of note, isoleucine was selected as part of the raw and residualized top-scoring pairs, which highlights that a single marker could be implicated in multiple reversals in marker ordering for disease states, depending on covariate adjustment.

**B Top 20 metabolite ions predictors from the LASSO and random forests**

Supplementary Table 1: Top 20 metabolite ions predictors from the LASSO and random forests methods, trained to the Chronic Renal Insufficiency Cohort (CRIC) Study sample of 977 participants with diabetes.

| LASSO | Random Forests | |
| --- | --- | --- |
| Raw | Raw | Residualized |
| Ion.4671 (0.56, 1.2)*  5-Aminolevulinic acid (0.53, 1.24) | Itaconate (4.64)* | Xanthine (2.16) |
|  | Dimethyl-Arg (4.17)* | Ion.7424 (1.87) ^†^ |
| Ornithine (0.43, 0.85)  12-Hydroxynevirapine (0.31, 0.71) | 5-Sulfosalicylic acid (3.64)* | Orotidine (1.43) |
| N-Methylcalystegine C1 (0.27, 0.84) | (Iso)Citrate (2.52) | (Iso)Leucine (1.28) |
|  | Xanthine (2.47) | dAMP (1.24) |
| Ion.3117 (0.22, 0.61)* | Aconitate (2.34) | Phenylalanine (1.18) |
| 3-Methyleneoxindole (0.21, 0.6) | Furoic acid (2.14)* | Ion.1554 (1.18) |
| Glutaminyltyrosine (0.21, 0.54)  Ion.4212 (0.19, 0.41)*  Acetylornithine (0.16, 0.37)  Mycophenolic acid (0.16, 0.32) | Ornithine (1.84) | Tryptophan (1.16) |
| Aminoadipate (0.15, 0.44) | Adenine (1.8) | Ion.1935 (1.15)* ^†^ |
|  | Methylguanine (1.71) | Biochanin A (1.11) |
|  | Butynal (1.7)* | Valine; Betaine (1.1)* |
| Carnosol (0.15, 0.37)  Trimethoprim (0.15, 0.3) | Ion.1935 (1.69)* ^†^ | Pipazethate (1.09) ^†^ |
|  | Valine; Betaine (1.64)* | Ornithine (1.09) |
|  | Ne,Ne dimethyllysine (1.63) | Ion.3778 (1.07) |
|  | Uracil (1.6) | Uracil (1.07) |
|  | Ion.2480 (1.47) | 5-Aminolevulinic acid (1.07) |
| Ion.2198 (0.15, 0.29) | Tetrose (1.44) | Neuraminic acid (1.04) |
|  | O-Demethylfonsecin (1.44)* | Nicotinurate (1.02) |
|  | 4-Nitrophenyl sulfate (1.3) | Ion.1884 (1.00) |
| Indoleacetyl glutamine (0.15, 0.21)  dAMP (0.14, 0.32)  Malate (0.13, 0.25)  5,6-Dihydrouridine (0.09, 0.34)  C10:3 (0.08, 0.24) | Ion.1554 (1.24) | Guaifenesin (0.99) |

LASSO, least absolute shrinkage and selection operator Values in () are expressed as effect size (standardized, unstandardized) for LASSO and mean decrease in Gini index from splitting on the metabolite ion, averaged over 500 trees, for random forests. *Selected as a raw metabolite ion predictor in K-TSP. ^†^Selected as a residualized metabolite ion predictor in K-TSP.

Ion.4671 – 13,14,15-trihydroxy-9-oxo-8,17-dioxatetracyclo[8.7.0.0²,⁷.0¹¹,¹⁶]heptadeca-1(10),2(7),3,5,11,13,15-heptaen-5-yl acetate

Ion.3117 – Asparaginyl-Hydroxyproline

Ion.4212 – 2-Phenylethyl beta-D-glucopyranoside

Ion.2198 – 6-Carboxy-5,6,7,8-tetrahydropterin

Ion.1935 – 3,6-Dihydro-4-(4-methyl-3-pentenyl)-1,2-dithiin

Ion.2480 – 3-(6-hydroxy-7-methoxy-2H-1,3-benzodioxol-5-yl)prop-2-enal

Ion.1554 – N-(3-acetamidopropyl)pyrrolidin-2-one

Ion.7424 – [4-(5-hydroxy-7-methoxy-8-methyl-4-oxo-4H-chromen-3-yl)-2-methoxyphenyl]oxidanesulfonic acid

Ion.1554 – N-(3-acetamidopropyl)pyrrolidin-2-one

Ion.3778 – 3-(6,7-dimethoxy-2H-1,3-benzodioxol-5-yl)oxirane-2-carboxylic acid

Ion.1884 – 5-Acetylamino-6-amino-3-methyluracil

**C Box plots of model prediction performance for DKD stage using the sample prevalence of patients with advanced stage DKD as the cutoff value**

Supplementary Figure 3: Box plots of model prediction performance for DKD stage: 100 repeats of 5-fold cross-validated (a) overall accuracy, (b) sensitivity, (c) specificity, (d) balanced accuracy, (e) positive predictive value, and (f) negative predictive value. Cutoff value for prediction in LASSO and random forests is the sample prevalence of patients with advanced stage DKD (0.205).

Model type:

(K-)TSP: (K) Top-Scoring Pair(s)

LASSO: Least Absolute Shrinkage and Selection Operator

RF: Random Forests
